# Influence of Different Saline and Alkali Aquaculture on the Nutritional Composition, Physicochemical Properties, and Flavor Profile of Grass Carp (*Ctenopharyngodon idellus*)

**DOI:** 10.3390/foods15183172

**Published:** 2026-09-08

**Authors:** Pengcheng Gao, Duanduan Yu, Peng Liu, Xinghe Chen, Long Li, Yanqing Huang, Qifang Lai, Hai Chi

**Affiliations:** 1 East China Sea Fisheries Research Institute, Chinese Academy of Fishery Sciences, Shanghai 200090, China; 2 Key Laboratory of Protection and Utilization of Aquatic Germplasm Resource, Liaoning Ocean and Fisheries Science Research Institute, Dalian 116023, China; 3 Jilin Fisheries Research Institute, Changchun 130022, China

**Keywords:** saline and alkali aquaculture, *Ctenopharyngodon idellus*, nutrition value, biochemical profile, comparative study

## Abstract

Saline and alkali aquaculture offers a sustainable strategy to alleviate pressure on freshwater resources and ecologically ameliorate saline and alkali environments; however, information regarding the quality of aquatic products from such conditions remains scarce. This study evaluated the nutritional components, color, texture, and flavor profiles of grass carp cultured under three representative saline and alkali conditions in China: carbonate (CB), chloride (CR), and sulfate (SF), with a freshwater-cultured grass carp (*Ctenopharyngodon idellus*) serving as a control (CK). The results obtained in our study showed that the CB group exhibited the lowest muscle water content (68.96 ± 0.04%). Crude fat and sodium levels were significantly elevated in all treatment groups compared to the CK group (*p* < 0.05). Conversely, the CK group displayed superior springiness and higher concentrations of geosmin, adenine, and uracil. Notably, the CR group exhibited the highest L* and whiteness values, whereas the CB and SF groups possessed significantly higher collagen content and enriched EPA and DHA levels. These findings demonstrate that saline and alkali conditions distinctly modulate the nutritional and flavor profiles of grass carp, highlighting their potential for producing value-added aquatic products under saline and alkali conditions.

## 1. Introduction

China possesses approximately 3.6 million hectares of saline–alkali land, accounting for 5% of the nation’s exploitable land area and spanning 17 provinces [1,2]. The majority of this land remains uncultivated due to challenges such as shallow groundwater levels with high mineralization and the accumulation of large concentrations of saline–alkali ions (Ca^2+^, Mg^2+^, K^+^, Na^+^, Cl^−^, CO_3_^2−^, and HCO_3_^−^) [3,4]. These factors hinder conventional agriculture and impede regional economic development.

Grass carp (*Ctenopharyngodon idellus*) is a key global freshwater species. Introduced to over 100 countries, it is cultivated outside China for affordable protein in nations such as Vietnam, Myanmar, and Afghanistan, and is widely used for biological macrophyte control in the US, India, Iran, and Europe [5]. Its rapid growth, broad environmental adaptability, and compatibility with polyculture systems support rural livelihoods, establishing it as a primary commercial species across diverse aquaculture systems [6]. Grass carp is characterized by its tender texture and mild flavor, coupled with a high content of protein and beneficial fatty acids. Its competitive price and superior nutritional density have already secured strong consumer acceptance in domestic markets. Beyond fresh consumption, grass carp exhibits significant potential as a raw material for processed aquatic products [7]. Compared to conventional species, the grass carp also offers distinct economic advantages due to its higher flesh yield, rapid growth rate, and lower production costs [8]. Given its wide availability and neutral taste profile, the grass carp serves as a sustainable feedstock capable of diversifying global raw material supply chains. The strategic utilization of this underutilized resource could reduce reliance on premium whitefish stocks and stimulate value-added innovation within the aquaculture processing industry.

Grass carp have been widely cultivated in China since the successful artificial reproduction of the “Four Famous Domestic Fishes” in 1958 [8]. Since then, grass carp farming in China has continuously increased. According to the Fishery Statistical Yearbook, the overall freshwater fish output in 2024 was 36.48 million tons, of which 7.41 million tons were produced by grass carp aquaculture alone [9]. With the rapid expansion of intensive aquaculture, the annual production of grass carp has increased; however, studies indicate that intensive farming has led to a deterioration in muscle texture and flavor (e.g., increased hardness but reduced juiciness and characteristic taste), suggesting a decline in overall eating quality [10]. In addition, feed and labor production costs continue to rise, and the market price of ordinary commercial grass carp has remained low for a long time. The advantages of grass carp aquaculture are declining [7]. Therefore, high-quality products are urgently required to address the homogeneity and low levels of grass carp farming in China. Recent studies have suggested that aquaculture environments significantly influence the nutritional composition of fish muscles; for instance, flowing-water and net cage systems yield different nutritional profiles compared to traditional pond-cultivated grass carp [10].

Saline and alkali aquaculture is increasingly being explored to address freshwater scarcity and land degradation, as it enables food production on marginal soils while simultaneously improving soil structure through leaching and reducing alkalinity [11]. Saline and alkali aquaculture in China has recently expanded successfully, improving soil quality and diversifying production [11,12]. Environmental factors, particularly salinity, are known to modulate the textural qualities of aquatic products, such as tilapia (*Oreochromis niloticus*) and rainbow trout [13,14]. Furthermore, changes in water salinity can impact not only the flavor of fish but also the distribution of their biochemical compositions. For example, largemouth bass (*Micropterus salmoides*) cultured in saline water exhibited higher EPA and DHA contents in their muscles than those raised in freshwater, and *Litopenaeus vannamei* cultivated in saline-salinity water improved amino acid profiles [15,16]. These findings imply that saline–alkali conditions may improve the flavor and nutritional value of fish, enhancing their market value. However, systematic studies on the quality of grass carp under saline and alkali conditions in China are rarely reported.

Therefore, this study systematically evaluated and compared the nutritional composition, amino acid and fatty acid profiles, and flavor compounds of grass carp cultivated under different saline and alkali conditions. Our findings provide compelling evidence for the utilization of saline and alkali lands to mitigate global food security challenges and freshwater scarcity. By bridging fundamental aquaculture science with global food industry priorities, these results advance both saline and alkali resource utilization and sustainable grass carp production.

## 2. Materials and Methods

### 2.1. Samples and Chemicals

Healthy three-year-old grass carp (average weight was 4.0 ± 1.5 kg) were selected from three distinct saline–alkali regions based on the natural distribution across China, where local geology and climate dictate the predominant saline and alkali chemistry: (A) Da’an City, Jilin Province (CB, carbonate type dominated by Na^+^ and CO_3_^2−^/HCO_3_^−^, E124°1′37.783″, N45°42′16.168″); (B) Tangshan City, Hebei Province (CR, chloride-type by Na^+^ and Cl^−^, E118°4′02.727”, N39°1′22.882″); and (C) Baiyin City, Gansu Province (SF, sulfate-type by Na^+^ and SO_4_^2−^, E104°18′20.963″, N37°11′29.314″) (Figure S1). Upon collection, the heads and tails were removed. The muscle was cut into thin slices (approximately 3–5 cm thick), vacuum-packed sterilely, and frozen at −16 °C for 1 h to ensure complete freezing. The samples were then transported to the laboratory with ice packs. The control group (CK) consisted of freshwater-cultured grass carp purchased from a supermarket in Dalian (Liaoning Province, China), which were acclimated to freshwater for 3 d prior to analysis. The salinity, alkalinity, and pH of all groups are detailed in Table 1.

All the chemicals used in the study, including CuSO_4_, H_2_SO_4_, NaOH, hydroxyproline, hydrochloric acid, phosphate buffer, and methanol were purchased from Sinopharm Chemical Reagent Co., Ltd. (Shanghai, China).

### 2.2. Proximate Composition Determination

The water content was determined using a water analyzer (Computrac MAX 4000XL, AMTEK-Brookfield, Middleborough, MA, USA). Crude protein, crude fat, and ash contents were analyzed using an automatic Kjeldahl apparatus (HGK-50, Kaidan, Shanghai, China), an automatic fat analyzer (YP-ZF10, Shandong Youyunpu Optoelectronic Technology Co., Ltd., Shanghai, China), and a rapid ash analyzer (HF-2, Hebi Xianfeng Instrument Co., Ltd., Hebi, China), respectively, following the method described by Huang et al. [17]. Crude protein content was calculated as N (g/100 g, dry basis) × 6.25.

### 2.3. Trace Element Analysis

The contents of eight trace elements, including sodium (Na), magnesium (Mg), potassium (K), calcium (Ca), iron (Fe), manganese (Mn), copper (Cu), and zinc (Zn) in grass carp were determined using the inductively coupled plasma mass spectrometry method (ICP-MS), in accordance with the method described by Hungerford et al. [18].

### 2.4. Water-Holding Capacity (WHC) and Steaming Loss Rate (SLR)

Approximately (3.00 ± 0.01) g of grass carp (marked as *M*_0_) was wrapped in filter paper and placed in a centrifuge tube. After centrifugation at 2240× *g* for 10 min, the weight of the sample was accurately measured (marked as *M*_1_). The water-holding capacity (WHC) of grass carp was calculated using Equation (1).

(1) $$WHC(\%)=\frac{1-(M_{0}-M_{1})}{M_{0}}\times 100\%$$

The steam loss rate (SLR) was measured according to the method described by Huang et al. [17]. Briefly, (10.00 ± 0.05) g of grass carp (marked as *M*_0_) was cut into pieces. The sample was then drained of excess water and placed in a tube. The sample was incubated in an 85 °C water bath for 15 min, after which the surface water was removed from the sample. The weight of the sample was accurately measured (marked as *M*_1_). The SLR was calculated using Equation (2).

(2) $$SLR(\%)=\frac{M_{0}-M_{1}}{M_{0}}\times 100\%$$

### 2.5. Texture Profile Analysis (TPA)

Grass carp back muscle cubes (approximately 15 mm × 15 mm × 15 mm) were carefully cut from the dorsal epaxial muscle using a stainless-steel scalpel, ensuring that the fiber direction was aligned vertically. All visible intermuscular bones were removed. The samples were allowed to equilibrate to room temperature for 30 min before analysis. The samples were measured using a P/36R probe (DIA Aluminum Radiused AACC, Stable Micro Systems Ltd., Surrey, UK) to conduct the TPA tests. Parameters, including hardness, elasticity, adhesiveness, gumminess, and chewiness, were measured at a pre-test speed of 2 mm/s, testing speed of 5 mm/s, post-test speed of 2 mm/s, and compression ratio of 50% with a holding time of 5 s during each measurement, based on preliminary tests and previous studies on fish texture [19].

### 2.6. Color Measurement

The color of the grass carp was determined using an automatic colorimeter (Chroma meter CR400, Konica, Tokyo, Japan). The color of the samples was represented by the parameters L*, a*, and b*. Whiteness (WI) is a measure of the lightness reflectivity of the fish meat surface, and the whiteness values are expressed in Equation (3). The colorimeter was calibrated against a white reference plate (CR-A43) and a zero-calibration cap prior to colorimetric measurements.

(3) $$WI=100-\sqrt{{\left(100-L^{*}\right)}^{2}+a^{*2}+b^{*2}}$$

### 2.7. Determination of Betaine and Taurine Contents

The betaine and taurine contents in grass carp muscle were determined according to the method described by Li et al. [20]. Briefly, approximately 2.0 ± 0.1 g of grass carp muscle tissue was homogenized in 10 mL of 0.1 mol/L HCl and centrifuged at 10,000× *g* for 15 min. The resulting supernatant was filtered through a 0.22 µm membrane. Betaine and taurine were analyzed by HPLC (Agilent 1260, Agilent Technologies, Inc., Santa Clara, CA, USA) on a Zorbax NH_2_ column (4.6 × 250 mm, 5 µm), using acetonitrile/water (80:20, *v/v*) as the mobile phase at a flow rate of 1.0 mL/min. Betaine was detected using an evaporative light scattering detector (ELSD), while taurine was quantified using a fluorescence detector (Ex 338 nm/Em 425 nm) following derivatization with o-phthaldialdehyde. External standards of betaine and taurine were used for quantification.

### 2.8. Determination of Collagen Content

Collagen content from the grass carp muscle was performed according to the instruction of the Solabio HYP (Beijing Solarbio Science & Technology Co., Ltd., Beijing, China) test kit. Briefly, approximately 2.0 ± 0.1 g of grass carp was cut into small pieces and placed in a glass tube. Approximately 2 mL of 6 mol/L hydrochloric acid solution was added to the sample, and then incubated in an oven at 110 °C for at least 2 h until no large clumps were visible within the glass tube. Approximately 0.3 mL of NaOH solution (10 mol/L) was used to adjust the pH of the sample to 6.0–8.0 after the sample cooled down, and the sample was subsequently transferred to a 10 mL volumetric flask and diluted to volume with distilled water. An aliquot of the supernatant (after centrifugation at 17,260× *g* for 10 min) was used for the assay. Approximately 1 mL of supernatant was diluted with 5 mL of distilled water. Subsequently, 2 mL of chloramine T solution was added, and the mixture was allowed to stand at room temperature for 20 min. Subsequently, the sample was combined with a chromogenic solution, sealed with foil, and incubated in a water bath at 60 °C for 30 min. Finally, the samples were measured at 560 nm. Distilled water and hydroxyproline standard solution (0.5 mg/mL) were used as the negative and positive controls, respectively. A standard curve was prepared using hydroxyproline standard solutions at concentrations of 0, 0.1, 0.2, 0.3, 0.4, and 0.5 mg/mL. The collagen content was calculated by multiplying the hydroxyproline concentration by a factor of 7.14, and expressed as μg/g of wet tissue.

### 2.9. Nucleotide Content Analysis

Approximately (0.1 ± 0.0) g of grass carp was mixed with 10 mL of 10% perchloric acid in a centrifuge tube and treated under ultrasonic conditions for 30 min. The mixture was centrifuged at 5460× *g* for 15 min, and the residue was mixed with 5% perchloric acid after the supernatant was removed. The supernatant was collected and adjusted to pH 6.5 with KOH, then diluted to 50 mL. After pH adjustment, the mixture was filtered through a 0.45 µm membrane to remove the precipitate. The residue was re-extracted with 5 mL of 5% perchloric acid, centrifuged again, and the combined supernatants were used for nucleotide analysis. Nucleotide determination was performed on 10 µL of the sample using the method of Li et al. [20]. Analyses were carried out on an Agilent 1260 HPLC system (Agilent Technologies Inc., Santa Clara, CA, USA) equipped with a diode array detector (DAD) and a C18 reversed-phase column (250 mm × 4.6 mm, 5 µm). The mobile phase consisted of (A) phosphate buffer and (B) 0.05 M methanol (A:B = 1000:40, *v/v*), delivered at a flow rate of 1 mL/min. Detection was performed at 254 nm. Standard solutions of ATP, ADP, AMP, IMP, GMP, CMP, and UMP (1.0 mg/mL, purchased from Sigma-Aldrich, St. Louis, MO, USA) were used for identification and quantification.

### 2.10. Amino Acid Analysis

Approximately (0.1 ± 0.0) g of grass carp was added to 5 mL of hydrochloric acid solution (6 mol/L) and placed into a tube for hydrolysis for 24 h at 110 °C. The hydrolyzate was filtered through a quantitative filter paper (Whatman Grade 1, pore size 11 µm), placed in a 10 mL flask, and diluted to the volume using distilled water. Subsequently, 0.05 mL of the filtrate was added to a 15 mL test tube and diluted with 2 mL of hydrochloric acid solution (0.02 mol/L) after drying with nitrogen. Approximately 20 μL of the sample, after passing through a 0.22 μm filter membrane, was used for amino acid content determination using the method described by Li et al. [20]. Chromatographic separation was performed on an ion-exchange column (4.6 mm × 60.0 mm, packed with ion-exchange resin) at 57 °C, with an injection volume of 20 µL and gradient elution. Post-column derivatization was carried out at 135 °C, and detection was performed at 570 nm (440 nm for proline). Tryptophan content was determined in accordance with the Chinese National Standard GB/T 15400-2018 [21].

### 2.11. Fatty Acid Analysis

The fatty acid content of grass carp was determined using the method described by Li et al. [20] via gas chromatography–mass spectrometry. Briefly, transesterification was performed by dissolving approximately 50 mg of the sample in 5 mL of 2.0% sulfuric acid in methanol within a fat extraction vial, followed by incubation at 70 °C for 60 min. The reaction mixture was subsequently quenched with 0.75 mL of ddH_2_O and 2 mL of n-hexane, followed by vigorous mixing. After stratification, the upper organic phase was carefully collected for fatty acid analysis. Fatty acid methyl esters were separated and quantified using a SCION-456-GC system (Beijing Timingtron Corporation, Beijing, China) equipped with a DB-23 capillary column (30 m × 320 µm × 0.25 µm, Agilent). The GC operating conditions were as follows: injection volume, 1 µL (split ratio 10:1); injector and flame ionization detector (FID) temperatures, 270 °C; carrier gas, high-purity nitrogen. The temperature gradient was programmed as follows: initial 130 °C (1 min), increased to 170 °C at 10 °C/min, then to 210 °C at 2.5 °C/min (final hold time 2 min). A 50 μL aliquot of C19 standard solution (1.0 mg/mL) was used as an internal standard for fatty acid content calculation according to Equation (4). The ambulatory index (IA) and thrombosis index (IT) are calculated using Equations (5) and (6), respectively, as proposed by Neri et al. [22].

(4) $$FAC\;(mg/g)=\frac{S_{x}\times 1.0\times 0.5}{S_{c19}\times m},$$

(5) $$Ambulatory\;Index,\;IA=\frac{C_{12:0\;}+C_{14:0}+C_{16:0}\;}{\sum PUMA+\sum MUFA},$$

(6) $$Thrombosis\;Index,\;IT=\frac{C_{12:0\;}+C_{14:0}+C_{16:0}\;}{0.5\times \sum PUMA+0.5\times \sum MUFA_{n-6}+3\times \sum PUFA_{n-3}+\frac{\sum PUFA_{n-3}}{\sum PUFA_{n-6}}},$$

where *S_x_* indicates the fatty acid peak area of the sample, *S_c_*_19_ indicates the fatty acid peak area of *C*_19_, and m indicates the sample weight.

### 2.12. Geosmin Analysis

Grass carp (0.5 ± 0.1 g) were added to 2 g of NaCl and 10 mL of distilled water, and the mixture was incubated for 30 min at room temperature. The sample was then transferred to a headspace solid-phase micro-extraction vial and stirred at 100× *g* at 60 °C for 30 min. The geosmin from grass carp was determined by using gas chromatography–mass spectrometry according to the method described by Tang et al. [23], with the following working program: chromatographic column: CB-5MS (30 m × 0.25 mm, 0.25 μm); temperature program: started at 50 °C, maintained for 1 min, then heated at a rate of 10 °C/min to 200 °C, maintained for 1 min, then heated at a rate of 20 °C/min to 220 °C, maintained for 1 min; initial temperature: 250 °C, initial pressure: 52 kPa, total flow rate: 44 mL/min, carrier gas: He.

### 2.13. Data Analysis

Each experiment was repeated at least three times in parallel, and the data were presented as “mean ± standard deviation”. Prior to parametric testing, normality was assessed using the Shapiro–Wilk test and homogeneity of variances was evaluated with Levene’s test. When both assumptions were met, one-way analysis of variance (ANOVA) was performed, followed by Tukey’s significant difference post hoc test for multiple comparisons. All statistical analyses were conducted using SPSS software (version 26.0), with *p* < 0.05 considered statistically significant.

## 3. Results

### 3.1. Proximate Composition

**Figure 1:**
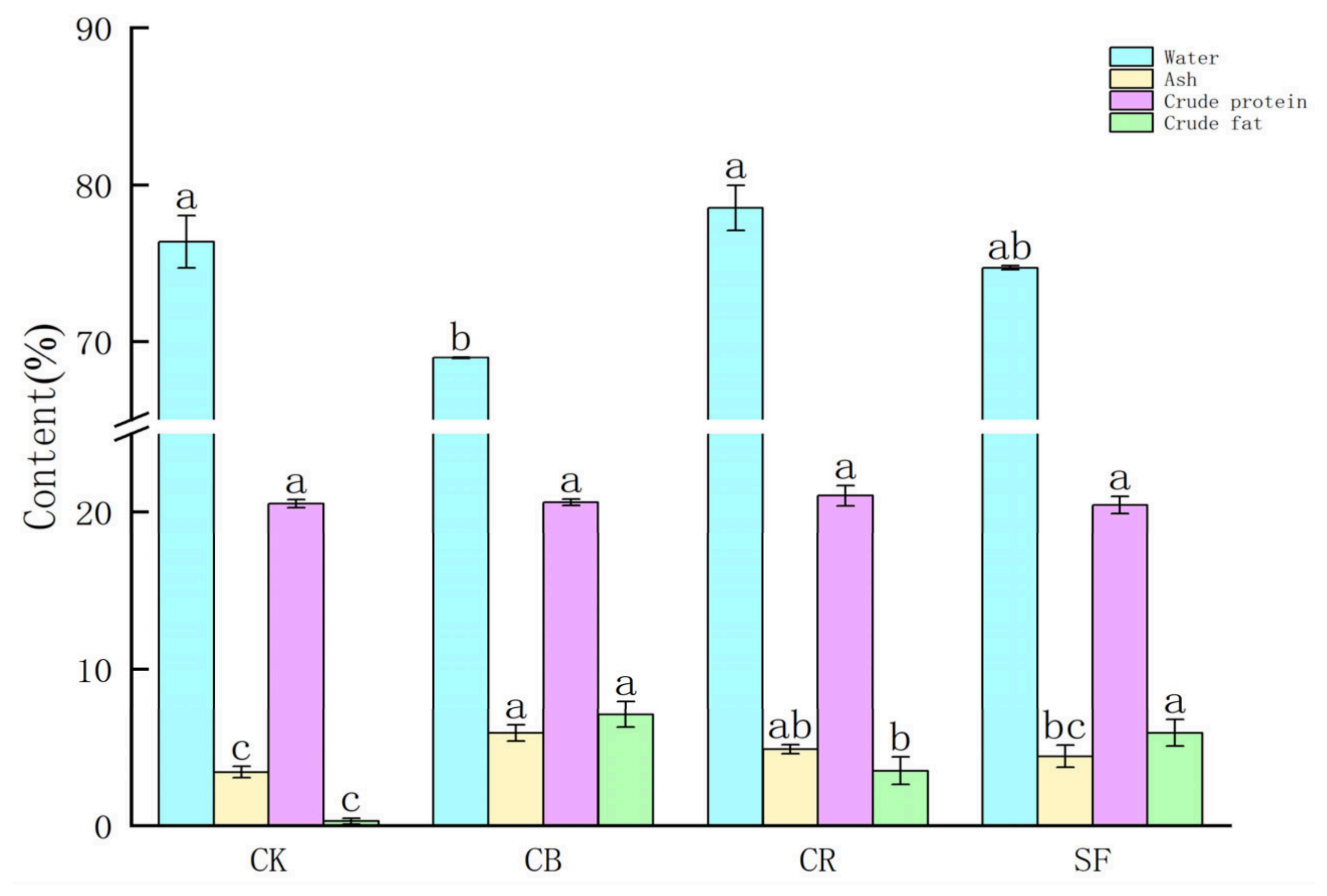
Contents of water, ash, crude fat and crude protein of grass carp under different saline and alkali conditions. Values are means ± SD (*n* = 3). Different letters on the bars within the same composition tested indicate significant differences (*p* < 0.05).

Figure 1 shows the proximate composition of grass carp under different saline and alkaline conditions. The CB group revealed the lowest water content (68.96 ± 0.04%). There was no significant difference in the water content between the grass carp in the CR and SF groups and the control group (*p* > 0.05). The ash content in the CB (5.91 ± 0.51%), CR (4.86 ± 0.29%), and SF (4.42 ± 0.70%) groups was significantly higher than that in the CK group (3.41 ± 0.36%) (*p* < 0.05). Crude fat content in the CK group (0.29 ± 0.19 g/100 g) was significantly lower than that in the experimental groups (*p* < 0.05). No significant differences in crude protein content were observed among the groups (*p* > 0.05). There was no significant difference in the crude protein content between the control and the three experimental groups (*p* > 0.05). The crude protein content was 20.52 ± 0.26% for CK, 20.62 ± 0.20 g/100 g for CB, 21.04 ± 0.65 g/100 g for CR, and 20.44 ± 0.55% for the SF group.

### 3.2. Trace Element Composition

Table 2 demonstrates the contents of the eight trace elements in grass carp under different saline–alkali conditions. The Na content varied significantly among the groups, with the highest level in CR (49.90 ± 0.33 g/100 g), followed by CB (42.20 ± 8.20 g/100 g) and SF (38.53 ± 0.15 g/100 g), and the CK group had the lowest Na content (27.40 ± 0.50 g/100 g). Mg and K contents in CK at values of 29.10 ± 1.00 g/100 g and 403.00 ± 9.00 g/100 g, respectively, differed significantly from those in CB and SF (*p* < 0.05). Ca content was highest in CR (11.33 ± 0.13 g/100 g). No significant differences were observed in the Fe and Zn contents. In addition, the Mn and Cu contents were below the detection limits in all samples.

### 3.3. Texture Profile Analysis

Table 3 shows the texture properties of grass carp under different saline–alkali conditions. The CK group exhibited significantly higher springiness (0.57 ± 0.04 mm) compared to the CB, SF, and CR groups (*p* < 0.05). The hardness values in the experimental groups (CB for 27.80 ± 3.03 N; CR for 27.36 ± 6.50 N; and SF for 26.47 ± 3.64 N) were significantly higher than those in CK (23.11 ± 2.86 N). Adhesiveness followed trends similar to those of hardness. The cohesiveness value in CK was 0.26 ± 0.01, showing no significant difference from that in the CB (0.20 ± 0.10). However, it differed significantly from the values in the CR (0.21 ± 0.02) and SF (0.20 ± 0.01) groups (*p* < 0.05). Additionally, there were no significant differences in gumminess and resilience between the CK and experimental groups (*p* > 0.05).

### 3.4. WHC and SLR

**Figure 2:**
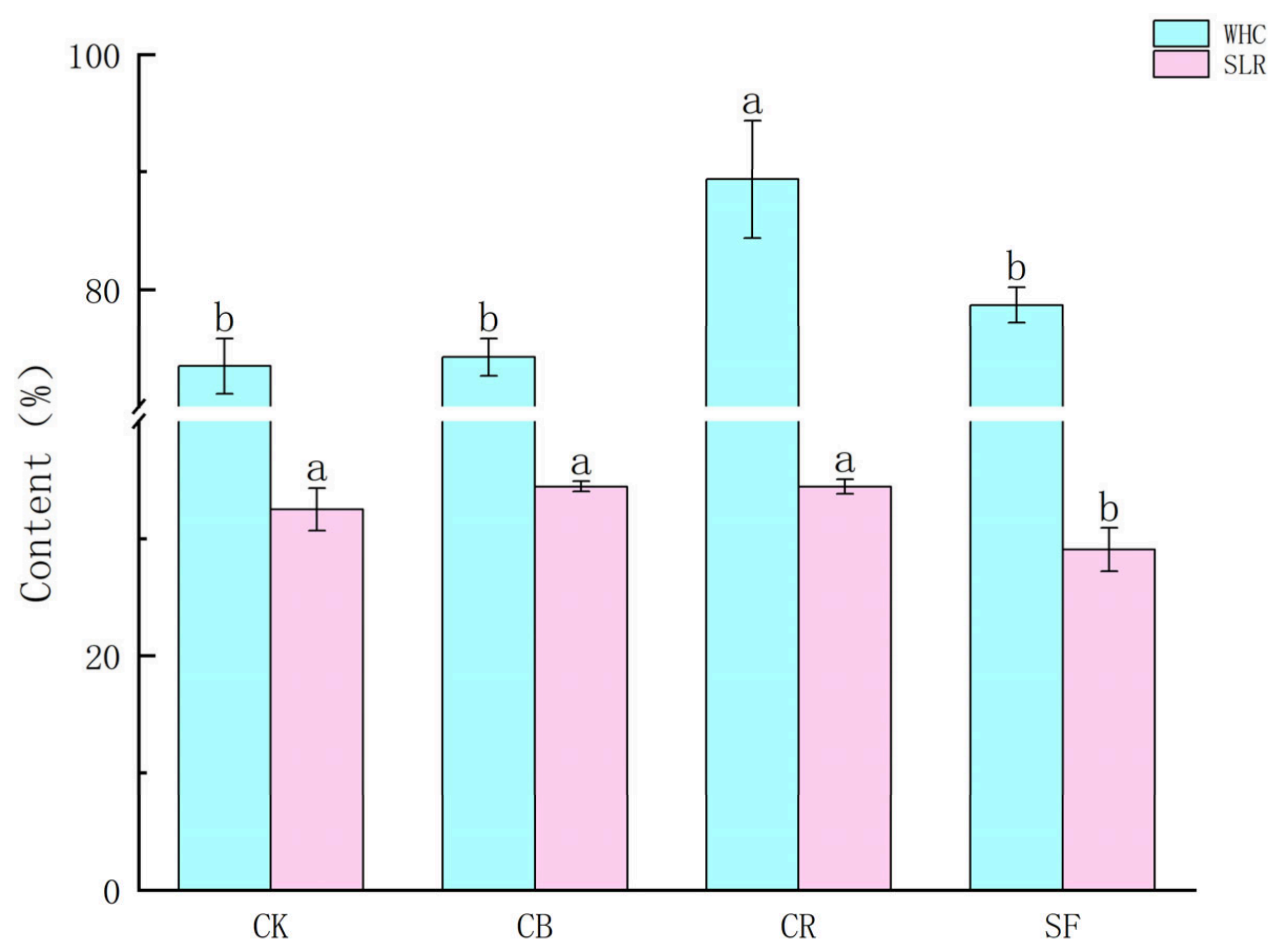
Water holding capacity (WHC) and steam loss rate (SLR) of grass carp under different saline and alkali conditions (wet basis). Values are means ± SD (*n* = 3). Different letters on the bars within the same composition tested indicate significant differences (*p* < 0.05).

Figure 2 shows the WHC and SLR of grass carp under different saline–alkali conditions. The results indicated that the CR group exhibited the highest WHC (89.36 ± 5.01%), significantly surpassing the other experimental groups (*p* < 0.05). In CK, as well as in the CB and SF groups, the WCHs were 73.44 ± 2.37%, 74.21 ± 1.60%, and 78.67 ± 1.52%, respectively. The SLR in the SF group (29.06 ± 1.84%) was significantly lower than that in the CK and other experimental groups (*p* < 0.05).

### 3.5. Color Analysis

Table 4 presents the color differences in grass carp under different saline and alkali conditions. The findings indicated that there were significant differences (*p* < 0.05) in the L* value, a* value, and WI of the grass carp between the CK and the three experimental groups. Among them, the CR group displayed the highest L* value (63.34 ± 0.94) and WI (63.27 ± 0.92), followed by the CB and SF groups, whereas the CK exhibited the lowest L* value and WI (42.55 ± 2.02 and 42.43 ± 2.02, respectively). The a* value of the grass carp was the highest (2.62 ± 0.87) in the CK group, showing significant differences (*p* < 0.05) compared to the other experimental groups. No significant differences (*p* > 0.05) were observed in the b* values of the grass carp among the groups.

### 3.6. Collagen Content

**Figure 3:**
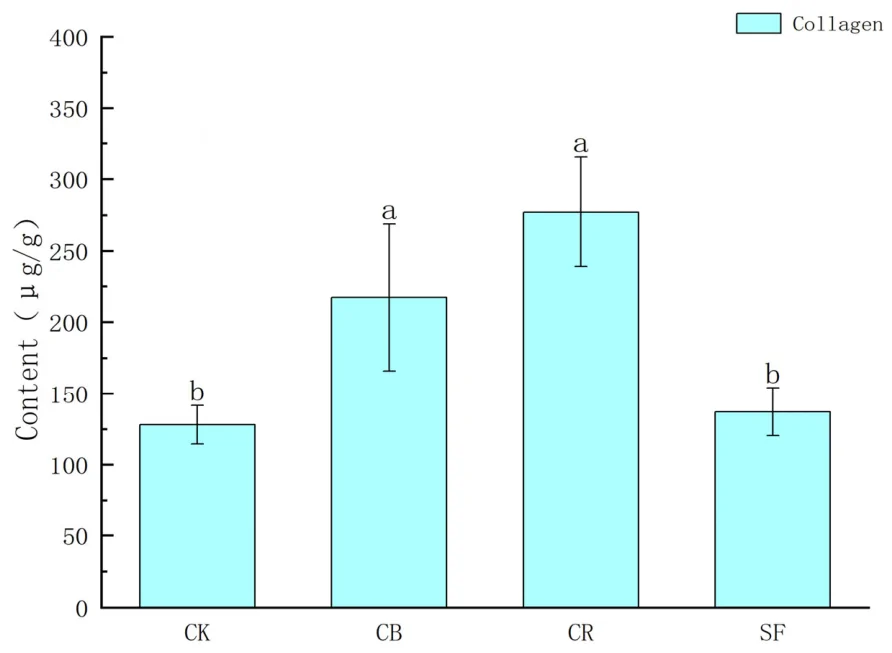
Collagen contents of grass carp under different saline and alkali conditions (wet basis). Values are means ± SD (*n* = 3). Different letters on the bars within the same composition tested indicate significant differences (*p* < 0.05).

Figure 3 shows the collagen content in grass carp under different saline–alkali conditions. The results indicated that the collagen contents in the CR and CB groups were significantly higher than those in the CK and CB groups, reaching 277.09 ± 38.45 μg/g and 217.36 ± 51.48 μg/g, respectively, compared to CK (128.13 ± 13.63 μg/g) (*p* < 0.05). The collagen content in the SF group was 137.21 ± 16.85 μg/g, which was slightly higher than that in the CK group, but not significantly different from that in the CK group (*p* > 0.05).

### 3.7. Nucleotide, Betaine, and Geosmin

Betaine acts as an osmolyte and flavor enhancer, contributing to sweetness and overall taste acceptance. Taurine is a functional amino acid associated with stress resistance and may influence the nutritional value of fish meat [17]. Table 5 shows the contents of betaine, taste-presenting nucleotides, and geosmin in grass carp under different saline and alkaline conditions. The betaine content in grass carp from the CB and CR groups was relatively high, at 20.32 ± 1.21 mg/kg and 11.21 ± 2.43 mg/kg, respectively, compared to that in the CK group (6.63 ± 0.02 mg/kg). The CMP, UMP, and IMP contents of grass carp in the CB group were significantly higher than those in the other groups, reaching 8.53 ± 1.02 mg/kg, 9.36 ± 0.60 mg/kg, and 221.31 ± 7.44 mg/kg, respectively. The geosmin, AMP, and UMP contents of the CK group were 6.69 ± 0.52 ng/g, 82.59 ± 5.86 mg/kg, and 12.39 ± 0.60 mg/kg, respectively, which were significantly higher than those in the three experimental groups (*p* < 0.05). In the SF group, only the contents of AMP and geosmin were significantly higher than those in the CB and CR groups, at 37.69 ± 3.40 mg/kg and 4.88 ± 0.14 ng/g, respectively, whereas the other contents were lower than those in grass carp from the CB and CR groups.

### 3.8. Amino Acid Profiles

Table 6 shows the content of amino acids of grass carp under different saline and alkali conditions. A total of 18 amino acids were detected in all grass carp samples, including eight essential amino acids (EAA) and 10 non-essential amino acids (NEAA). Among these amino acids, the highest content was glutamic acid (Glu), which has an important relationship with food freshness. The content of Glu in the CK group was 2.28 ± 0.12 g/100 g, while in the experimental group, it was 2.18 ± 0.16 g/100 g in the CB and 2.26 ± 0.12 g/100 g in the SF groups. In contrast, the content in the experimental group in CR group was lower, only 1.22 ± 0.07 g/100 g. The second highest content was aspartic acid (Asp) and leucine (Leu). The content of Asp in the CK group was 1.65 ± 0.06 g/100 g, while in the experimental group, it was 1.53 ± 0.07 g/100 g in the CB and 1.72 ± 0.08 g/100 g in the CR groups. The content in the experimental group in CR groups was the lowest, only 0.82 ± 0.04 g/100 g. The content of Leu in the CK group was 1.37 ± 0.09 g/100 g, while in the experimental group, it was 1.21 ± 0.08 g/100 g in the CB and 1.45 ± 0.08 g/100 g in the SF groups. The content in the CR group was 0.69 ± 0.03 g/100 g. Among these amino acids, cysteine (Cys) had the lowest content. The Cys content in the CK group was only 0.08 ± 0.00 g/100 g, while in other experimental groups it was 0.09 ± 0.01 g/100 g in the CB and 0.07 ± 0.01 g/100 g in the SF groups. However, no Cys content was detected in the CR group.

### 3.9. Fatty Acid Profiles

In total, 12 saturated fatty acids (SFAs), 8 monounsaturated fatty acids (MUFAs), and 14 polyunsaturated fatty acids (PUFAs) were detected in the muscles of grass carp cultured under different saline and alkali conditions (Table 7). Among them, C14:0, C16:0, C16:1n7, C18:1n9, C18:2n6, C20:5n3 (EPA), and C22:6n3 (DHA) constituted the main SFAs, MUFAs, and PUFAs, respectively. In the grass carp from the CB and SF groups, as well as in the CK group, the content of C18:1n9 was the highest, at 38.20 ± 4.18 mg/g, 25.83 ± 2.42 mg/g, and 54.03 ± 9.52 mg/g, respectively. In the CK group, the C14:0 content was lower than that in the CB and SF groups but higher than that in the CR group. Similarly, C16:1n7 and C22:6n3 exhibited significant differences (*p* < 0.05). The C20:4n6 content in the CR group was 7.43 ± 1.04 mg/g, which was significantly higher than that in the other three groups (*p* < 0.05). The EPA and DHA contents in the CB and SF groups were significantly higher than those in the CK group (*p* < 0.05). Specifically, in the experimental group from the CR group, the content of EPA + DHA was 2.57 ± 0.32 mg/g.

In addition, the IA of the CK group was 0.27, while the values for the CB, CR, and SF groups were 0.35, 0.34, and 0.43, respectively. The SF group showed the highest IA, indicating a relatively higher potential to promote atherosclerosis than the other groups. The thrombosis index (IT) was 0.42 in the CK group. In contrast, the IT values for the samples from the CB, CR, and SF groups were 0.25, 0.38, and 0.36, respectively. The CB group exhibited the lowest IT, suggesting a relatively lower thrombotic potential, whereas the CR group showed the highest IT among the experimental groups.

## 4. Discussion

This study presents the first comprehensive comparative analysis of how distinct saline and alkali types, including carbonate (CB), chloride (CR), and sulfate (SF), modulate the muscle quality of grass carp in China. Our findings demonstrate that saline and alkali aquaculture transcends the mere mitigation of land resource constraints and represents a viable strategy for quality differentiation in freshwater aquaculture. Although traditional pond and cage systems yield notable variations in nutritional quality, diverse species, including tilapia, *Litopenaeus vannamei*, *Macrobrachium rosenbergii*, and *Micropterus salmoides*, have been successfully cultivated under saline–alkali conditions [13,16,24,25]. This diversification offers substantial potential for saline and alkali utilization, farmer income growth, and the enhancement of aquatic product quality.

Na^+^ is the primary determinant of saltiness in grass carp muscle. During brine acclimation or salting, enhanced NaCl penetration raises Na^+^ levels in the sarcoplasm, directly intensifying the salty flavor profile while interacting synergistically with umami compounds (IMP) and free amino acids (Glu/Asp) to elevate palatability [26]. Na^+^ also facilitates deeper salt diffusion into myofibrillar proteins and triggers mild lipid oxidation, producing the specific aldehydes and ketones desirable in dry-cured grass carp [27]. Conversely, sulfate (SO_4_^2−^) does not contribute directly to taste at these concentrations. Instead, sulfate exerts a metabolic effect, shifting the metabolism of sulfur-containing amino acids (Met and Cys) and their volatile derivatives. While the distinct flavor impact of SO_4_^2−^ remains unquantified in grass carp, it likely acts indirectly by modulating sulfur precursor pools to influence Maillard reactions and sulfurous aromas during thermal processing [28].

The results provided subtle yet significant variations in the proximate composition, texture, color, amino acid profiles, and fatty acid profiles of grass carp across the three typical saline–alkali conditions relative to the freshwater control. Consistent with Jia [7] and Zhang’s reports on salinity-mediated flavor modulation, our results confirmed that saline–alkali conditions distinctly influenced fish muscle quality [26]. Specifically, grass carp from the CR group exhibited superior hardness and WHC, along with the lowest CLR, significantly outperforming the CK (*p* < 0.05). These improvements are likely attributable to the formation of a gel-like network structure in myofibrillar proteins (particularly myosin) under high-salinity conditions [29]. This structure enhances the structural integrity while effectively immobilizing water within its porous matrix. Similarly, Huang et al. [17] reported increased meat yield and WHC in crayfish under saline–alkali conditions. The underlying mechanism may involve competitive inhibition by elevated sodium ion levels, which disrupt potassium absorption and storage. Given the role of potassium in maintaining myofibrillar protein structure, its depletion likely alters fiber morphology and reduces water exudation [30]. Furthermore, grass carp in the CR group showed the most pronounced alterations in collagen content, L* value, WI, and geosmin levels (*p* < 0.05), aligning with the observations of Wang [31] and Zhang [26] regarding the positive impact of salinity on fish quality and flavor.

From a physiological perspective, saline stress in grass carp represents a primary osmoregulatory challenge. Acute exposure to 4–10 ppt elevated serum Na^+^, Cl^−^, osmolality, and cortisol, while upregulating gill Na^+^/K^+^-ATPase and SLC12A2 (NKCC) expression [32]. Moderate salinity (3–5‰) is beneficial to fillet quality; the muscle fibers narrow, the sarcolemma thickens, the hydroxyproline and collagen-related gene expressions rise, and texture attributes (hardness, springiness, chewiness) improve relative to freshwater controls. The WHC gain is linked to mild osmotic dehydration balanced by myofibrillar protein hydration and reduced extracellular space. However, higher salinities (>7–9‰) induce the osmoregulatory response; the resulting chronic Cl^−^/SO_4_^2−^ load depresses growth, lowers collagen, texture, and reduces WHC. Although sulfate increases ionic strength, NaCl osmolality is the principal driver of textural changes, whereas sulfate primarily influences sulfur-amino-acid metabolism [26].

In contrast to the CB group, which displayed the highest muscular IMP content and lowest adenine levels (*p* < 0.05), the hepatic response to chronic high alkalinity revealed a systemic energy metabolism [33,34]. High-alkalinity stress imposes a severe osmoregulatory burden and respiratory acidosis, driving ATP depletion as resources are diverted to ion pumping and ammonia detoxification via the Glu-Gln pathway. This energy deficit activates AMPK, which facilitates AMP conversion to IMP while suppressing de novo purine synthesis, explaining the accumulation of IMP and the reduction of adenine nucleotides observed in the muscle [35,36]. At the mechanistic level, transcriptomic and metabolomic data have shown that suppressed oxidative phosphorylation and activated purine degradation create a metabolic bottleneck. Consequently, alkalinity-induced stress activates the classic purine catabolism pathway, driving ATP exhaustion, AMP deamination, and subsequent IMP accumulation. This process is triggered by ammonia toxicity and metabolic reprogramming rather than post-mortem hypoxia [37]. Future studies should quantify AMPK activity, track purine metabolic fluxes, and integrate transcriptomics to clarify how chronic alkalinity reprograms energy allocation in the grass carp muscles.

Finally, grass carp in the SF group exhibited the highest concentrations of EAA and PUFAs, particularly DHA and EPA (*p* < 0.05). This dual enhancement likely reflects comprehensive physiological adaptations to osmotic stress [24,38,39]. To maintain cellular homeostasis, fish accumulate osmolytes to stabilize enzyme activity and cell structure, contributing directly to the elevation of amino acid reserves. Additionally, the preferential synthesis of stress-resistant proteins (e.g., antioxidants and osmoregulators) increases muscle amino acid pools [38,40]. Future studies, therefore, should trace PUFA biosynthesis pathways and identify key enzymes regulating EAA allocation, while validating how these metabolites bolster membrane resilience under different saline–alkali stress. To counteract the membrane rigidity induced by high salinity and oxidative stress, grass carp enhance the proportion of long-chain PUFAs in muscle cell membranes to preserve fluidity and selective permeability [41]. Analogous trends have been documented in Japanese seabass (*Lateolabrax japonicus*) under varying environmental conditions [42].

## 5. Conclusions

This study demonstrated that saline–alkali aquaculture significantly alters the quality traits of grass carp. Compared to freshwater controls, saline and alkali conditions resulted in increased muscle ash, crude fat, and sodium content. Texturally, the grass carp in the chloride-type (CR) group exhibited improved hardness and WHC. Crucially, the color (particularly WI) was enhanced, and the off-flavor compounds (geosmin) were reduced. Furthermore, specific conditions conferred unique nutritional benefits, such as carbonate-type waters (CB) promoting IMP accumulation, whereas sulfate-type waters (SF) significantly enhanced EPA, DHA, and EAA contents. These findings underscore the viability of saline and alkali aquaculture as a strategy for producing differentiated, high-quality grass carp products. Future studies should focus on elucidating the molecular mechanisms governing these physiological adaptations and optimizing farming protocols based on the target quality traits.

## Figures and Tables

**Table 1:** Salinity, alkalinity, and pH values of control group and three different regions where grass carp were collected.

	Salinity (‰)	Alkalinity (mmol/L)	pH Values
CB	0.75 ± 0.01	10.32 ± 0.23	8.72 ± 0.03
CR	6.44 ± 0.05	14.31 ± 0.15	8.78 ± 0.02
SF	5.03 ± 0.02	3.84 ± 0.13	7.72 ± 0.00
CK	0.09 ± 0.01	1.18 ± 0.27	7.11 ± 0.10

Note: Values are expressed as mean ± standard deviation (SD), *n* = 3.

**Table 2:** Content of 8 trace elements in grass carp under different saline and alkali types (dry basis, g/100 g).

	CK	CB	CR	SF
Na	27.40 ± 0.50 ab	42.20 ± 8.20 c	49.90 ± 0.33 a	38.53 ± 0.15 b
Mg	29.10 ± 1.00 b	25.43 ± 1.68 a	26.60 ± 0.00 b	30.38 ± 0.12 a
K	403.00 ± 9.00 b	356.00 ± 2.00 a	362.00 ± 0.00 b	409.89 ± 2.78 a
Ca	7.60 ± 0.18 a	9.54 ± 0.30 a	11.33 ± 0.13 a	10.83 ± 0.23 a
Fe	0.41 ± 0.01 a	0.36 ± 0.01 a	0.49 ± 0.07 a	0.30 ± 0.01 a
Mn	<0.03	<0.03	<0.03	<0.03
Cu	<0.02	<0.02	<0.02	<0.02
Zn	0.37 ± 0.01 a	0.41 ± 0.04 a	0.39 ± 0.00 a	0.46 ± 0.01 a

Note: Values are expressed as mean ± standard deviation (SD), *n* = 3. Different letters in the same row indicate significant differences (*p* < 0.05).

**Table 3:** TPA results of grass carp under different saline and alkali conditions.

	CK	CB	CR	SF
Hardness (N)	23.11 ± 2.86 b	27.80 ± 3.03 a	27.36 ± 6.50 a	26.47 ± 3.46 a
Adhesiveness (g)	6.94 ± 0.98 b	10.44 ± 1.38 a	11.57 ± 1.50 a	9.88 ± 0.76 a
Springiness (mm)	0.57 ± 0.04 a	0.42 ± 0.01 b	0.47 ± 0.03 b	0.46 ± 0.02 b
Cohesiveness	0.26 ± 0.01 ab	0.20 ± 0.10 a	0.21 ± 0.02 b	0.20 ± 0.01 b
Gumminess (g)	614.26 ± 65.91 a	368.26 ± 46.15 a	592.79 ± 121.70 a	559.90 ± 93.63 a
Chewiness (mJ)	350.88 ± 28.94 a	155.90 ± 18.92 c	293.64 ± 66.50 b	355.51 ± 61.74 a
Resilience	0.12 ± 0.01 a	0.10 ± 0.01 a	0.13 ± 0.03 a	0.09 ± 0.01 a

Note: Values are expressed as mean ± standard deviation (SD), *n* = 3. Different letters in the same row indicate significant differences (*p* < 0.05).

**Table 4:** Color of grass carp under different saline–alkali conditions.

	CK	CB	CR	SF
L*	42.55 ± 2.02 c	49.10 ± 0.61 b	63.34 ± 0.94 a	48.00 ± 2.21 b
a*	2.62 ± 0.87 a	0.64 ± 0.12 b	−1.59 ± 0.43 c	−1.20 ± 0.44 c
b*	2.17 ± 0.99 a	0.88 ± 0.33 a	0.67 ± 1.34 a	1.90 ± 1.43 a
WI	42.43 ± 2.02 c	49.08 ± 0.61 b	63.27 ± 0.92 a	48.03 ± 2.21 b

Note: Values are expressed as mean ± standard deviation (SD), *n* = 3. Different letters in the same row indicate significant differences (*p* < 0.05).

**Table 5:** Contents of nucleotide, betaine, and geosmin of grass carp under different saline and alkali conditions (wet basis, ng/g for geosmin, and mg/kg for the remaining testing indexes).

	CK	CB	CR	SF
CMP	5.13 ± 0.26 b	8.53 ± 1.02 c	2.02 ± 0.34 a	1.37 ± 0.06 a
UMP	12.39 ± 0.60 c	9.36 ± 0.60 b	5.27 ± 0.74 a	<0.2
AMP	82.59 ± 5.86 d	4.82 ± 0.82 a	19.52 ± 1.51 b	37.69 ± 3.40 c
GMP	2.46 ± 0.32 b	5.37 ± 0.61 c	1.48 ± 0.08 a	<0.52
IMP	4.54 ± 0.14 a	221.31 ± 7.44 c	17.83 ± 1.82 b	11.57 ± 0.95 b
Betaine	6.63 ± 0.02 b	20.32 ± 1.21 d	11.21 ± 2.34 c	0.31 ± 0.12 a
Geosmin	6.69 ± 0.52 d	2.63 ± 0.66 b	1.32 ± 0.52 a	4.88 ± 0.14 c

Note: Values are expressed as mean ± standard deviation (SD), *n* = 3. Different letters in the same row indicate significant differences (*p* < 0.05).

**Table 6:** Contents of amino acids of grass carp under different saline and alkali conditions (dry basis, g/100 g).

	CK	CB	CR	SF
Tau	0.89 ± 0.04 b	0.60 ± 0.07 c	1.02 ± 0.02 a	0.60 ± 0.04 c
Asp	1.65 ± 0.06 a	1.53 ± 0.07 a	0.82 ± 0.04 b	1.72 ± 0.08 a
Thr	0.71 ± 0.04 a	0.68 ± 0.10 a	0.37 ± 0.02 b	0.74 ± 0.05 a
Ser	0.56 ± 0.08 a	0.58 ± 0.08 a	0.32 ± 0.09 b	0.57 ± 0.07 a
Glu	2.28 ± 0.12 a	2.18 ± 0.16 a	1.22 ± 0.07 b	2.26 ± 0.12 a
Gly	0.71 ± 0.02 b	0.75 ± 0.06 b	0.72 ± 0.00 b	0.87 ± 0.10 a
Ala	0.97 ± 0.01 a	0.89 ± 0.02 b	0.59 ± 0.06 c	1.04 ± 0.08 a
Cys	0.08 ± 0.00 a	0.09 ± 0.01 a	<0.02	0.07 ± 0.01 a
Val	0.93 ± 0.04 a	0.82 ± 0.05 b	0.45 ± 0.03 c	0.98 ± 0.03 a
Met	0.35 ± 0.06 a	0.22 ± 0.04 b	0.07 ± 0.01 c	0.32 ± 0.02 a
IIe	0.79 ± 0.02 b	0.70 ± 0.04 b	0.41 ± 0.05 c	0.88 ± 0.06 a
Leu	1.37 ± 0.09 b	1.21 ± 0.08 b	0.69 ± 0.03 c	1.45 ± 0.08 a
Tyr	0.56 ± 0.07 a	0.46 ± 0.06 b	0.27 ± 0.04 c	0.54 ± 0.04 a
Phe	0.68 ± 0.06 b	0.64 ± 0.05 b	0.35 ± 0.02 c	0.83 ± 0.12 a
Trp	0.82 ± 0.05 a	0.84 ± 0.05 a	0.84 ± 0.03 a	0.85 ± 0.03 a
Lys	1.63 ± 0.08 b	1.45 ± 0.09 b	0.78 ± 0.03 c	1.78 ± 0.04 a
His	0.43 ± 0.04 b	0.44 ± 0.03 b	0.17 ± 0.02 c	0.55 ± 0.01 a
Arg	0.97 ± 0.01 a	0.90 ± 0.02 a	0.61 ± 0.02 b	1.03 ± 0.07 a
Pro	0.50 ± 0.00 a	0.53 ± 0.00 a	0.46 ± 0.05 a	0.52 ± 0.01 a
EAA	7.48 ± 0.16 a	6.56 ± 0.08 b	3.96 ± 0.16 c	7.83 ± 0.03 a
NEAA	8.71 ± 0.09 b	8.35 ± 0.10 b	5.18 ± 0.07 c	9.17 ± 0.15 a
TAA	16.19 ± 0.20 a	14.91 ± 0.28 b	9.14 ± 0.06 c	17.00 ± 0.82 a
EAA/TAA (%)	46.20 ± 0.08 a	46.52 ± 0.23 a	43.33 ± 0.43 b	46.05 ± 0.17 a
EAA/NEAA (%)	85.88 ± 1.93 a	78.56 ± 0.25 b	76.45 ± 0.02 c	85.39 ± 2.01 a

Note: Values are expressed as mean ± standard deviation (SD), *n* = 3. Different letters in the same row indicate significant differences (*p* < 0.05).

**Table 7:** Contents of fatty acids of grass carp under different saline and alkali conditions (dry basis, mg/g).

	CK	CB	CR	SF
C4:0	0.39 ± 0.24 a	0.01 ± 0.01 b	0.04 ± 0.04 b	0.03 ± 0.01 b
C6:0	0.25 ± 0.06 a	0.23 ± 0.05 ab	0.09 ± 0.09 b	0.13 ± 0.08 ab
C8:0	0.04 ± 0.02 ab	0.08 ± 0.02 ab	0.03 ± 0.03 ab	0.02 ± 0.01 b
C10:0	0.03 ± 0.02 a	0.03 ± 0.01 a	0.02 ± 0.01 a	0.01 ± 0.01 a
C12:0	0.03 ± 0.02 b	0.10 ± 0.02 a	0.02 ± 0.01 b	0.10 ± 0.03 a
C13:0	0.00 ± 0.00 a	0.02 ± 0.00 a	0.00 ± 0.00 a	0.02 ± 0.01 a
C14:0	1.11 ± 0.10 b	1.92 ± 0.56 a	0.21 ± 0.04 c	2.20 ± 0.45 a
C15:0	0.17 ± 0.01 a	0.52 ± 0.11 a	0.07 ± 0.02 a	0.49 ± 0.11 a
C16:0	21.63 ± 0.92 a	24.01 ± 4.56 a	6.89 ± 0.44 a	30.41 ± 5.10 a
C17:0	0.00 ± 0.00 a	0.74 ± 0.15 a	0.10 ± 0.05 a	0.47 ± 0.09 a
C18:0	0.00 ± 0.00 a	0.00 ± 0.00 a	0.00 ± 0.00 a	6.08 ± 3.46 a
C20:0	0.17 ± 0.01 a	0.14 ± 0.11 a	0.16 ± 0.04 b	0.16 ± 0.07 a
SFAs	23.82 ± 1.40	27.8 ± 5.60	7.63 ± 0.77	40.12 ± 9.43
C14:1	0.02 ± 0.01 ab	0.07 ± 0.04 a	0.00 ± 0.00 a	0.08 ± 0.05 a
C16:1n9	0.55 ± 0.28 a	0.80 ± 0.18 a	0.07 ± 0.03 a	0.80 ± 0.19 a
C16:1n7	4.28 ± 0.23 b	7.34 ± 1.96 a	0.45 ± 0.16 b	9.56 ± 1.82 a
C18:1n9t	0.10 ± 0.05 a	0.00 ± 0.00 a	0.00 ± 0.00 a	0.25 ± 0.18 a
C18:1n9	38.20 ± 4.18 b	25.83 ± 2.42 c	4.90 ± 1.36 d	54.03 ± 9.52 a
C18:1n7	2.70 ± 0.27 a	2.34 ± 0.53 b	0.31 ± 0.23 b	3.82 ± 0.56 b
C20:1	0.70 ± 0.01 b	0.65 ± 0.07 b	0.16 ± 0.04 c	1.30 ± 0.24 a
MUFAs	46.6 ± 5.05	37.23 ± 5.23	5.08 ± 1.82	70.03 ± 12.57
C16:2n6	1.04 ± 0.77 a	0.16 ± 0.04 a	0.00 ± 0.00 a	0.24 ± 0.08 a
C16:3n6	0.00 ± 0.00 a	0.03 ± 0.03 a	0.00 ± 0.00 a	0.02 ± 0.02 a
C16:2n4	0.14 ± 0.01 a	0.50 ± 0.13 a	0.00 ± 0.00 a	0.68 ± 0.18 a
C16:4n3	0.01 ± 0.00 a	0.07 ± 0.04 a	0.08 ± 0.00 a	0.03 ± 0.03 a
C18:2n6	24.66 ± 3.95 a	11.55 ± 0.50 b	4.69 ± 0.67 c	20.19 ± 3.54 a
C18:3n3	1.90 ± 0.32 bc	14.59 ± 3.62 a	0.54 ± 0.15 c	4.97 ± 1.88 b
C18:4n3	0.00 ± 0.00 a	0.25 ± 0.16 a	0.00 ± 0.00 a	0.16 ± 0.06 ab
C20:2n6	1.15 ± 0.16 b	0.86 ± 0.06 c	0.24 ± 0.12 d	1.61 ± 0.20 a
C20:3n6	1.20 ± 0.20 b	0.67 ± 0.05 c	0.50 ± 0.13 c	1.52 ± 0.15 a
C20:4n6	3.97 ± 0.57 b	2.10 ± 0.26 c	7.43 ± 1.04 a	4.07 ± 0.33 b
C20:3n3	0.01 ± 0.00 c	1.26 ± 0.22 a	0.00 ± 0.00 a	0.41 ± 0.19 b
C20:4n3	0.00 ± 0.00 a	0.72 ± 0.19 a	0.00 ± 0.00 a	0.49 ± 0.13 b
C20:5n3	0.32 ± 0.01 b	1.80 ± 0.23 a	0.25 ± 0.16 b	1.99 ± 0.18 a
C22:6n3	2.47 ± 0.44 c	3.65 ± 0.59 b	2.32 ± 0.26 c	5.92 ± 0.54 a
PUFAs	36.87 ± 6.63	38.21 ± 6.07	16.05 ± 2.53	42.3 ± 7.51
DPA+DHA	2.79 ± 0.43	5.45 ± 0.82	2.57 ± 0.32	7.91 ± 0.69
IA	0.27	0.35	0.34	0.43
IT	0.42	0.25	0.38	0.36

Note: Values are expressed as mean ± standard deviation (SD), *n* = 3. Different letters in the same row indicate significant differences (*p* < 0.05).

## Data Availability

The raw data supporting the conclusions of this article will be made available by the authors upon request.

## Supplementary Materials

The following supporting information can be downloaded at https://www.mdpi.com/article/10.3390/foods15183172/s1, Figure S1: Illustration of sample collection sites from different saline and alkali conditions.
