# Supplementary material for: Influence of Different Saline and Alkali Aquaculture on the Nutritional Composition, Physicochemical Properties, and Flavor Profile of Grass Carp (Ctenopharyngodon idellus)
Source: Foods. 2026 Sep 8;15(18):3172. doi: 10.3390/foods15183172 (PMC13604979; doi:10.3390/foods15183172)
Supplement: Supplementary file 1 [file foods-15-03172-s001.zip › foods-4514520-supplementary.pdf]

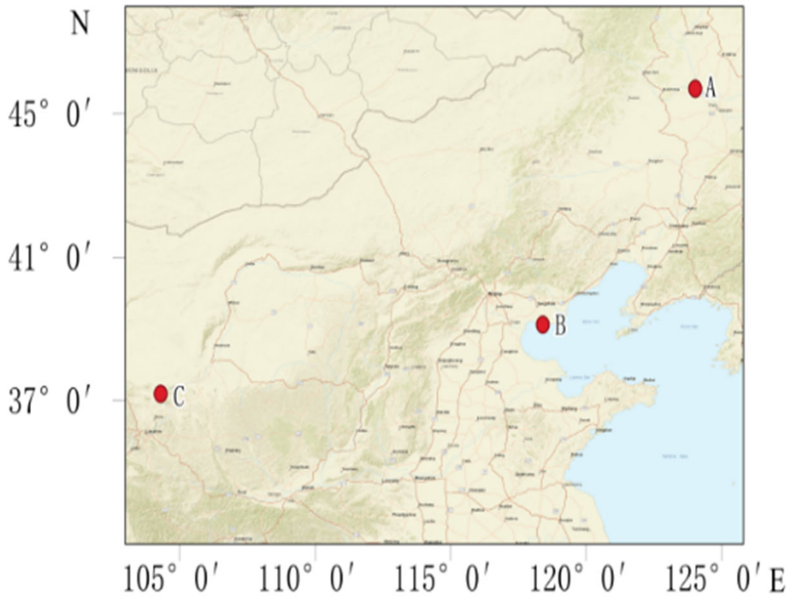

Figure S1. Illustration of sample collection sites from different saline and alkali conditions. Note: A is located in Da’an City, Jilin Province; B is located in Tangshan City, Hebei Province; and C is located in Baiyin City, Gansu Province. They represent the carbonate, chloride, and sulfate types for A, B, and C, respectively.
